# De omisiones especializadas: la biomedicina como parte intrínseca de la vida de los pueblos originarios

**DOI:** 10.18294/sc.2023.4539

**Published:** 2023-08-11

**Authors:** Eduardo L. Menéndez

**Affiliations:** 1 Doctor en Ciencias Antropológicas. Doctor Honoris Causa, Universitat Rovira i Virgili, Catalunia; Doctor Honoris Causa, Universidad Nacional de Rosario, Argentina; Doctor Honoris Causa, Universidad Nacional de Lanús, Argentina. Profesor-investigador emérito, Centro de Investigaciones y Estudios Superiores en Antropología Social (CIESAS), México. emenendez1@yahoo.com.mx Universidad Nacional de Lanús Universidad Nacional de Lanús Argentina emenendez1@yahoo.com.mx; Universitat Rovira i Virgili Catalunia España; Universidad Nacional de Rosario Argentina; Centro de Investigaciones y Estudios Superiores en Antropología Social (CIESAS) MEXICO

**Keywords:** Antropología Médica, Pueblos Indígenas, Medicina Tradicional, Biomedicina, Medical Anthropology, Indigenous Peoples, Traditional Medicine, Biomedicine

## Abstract

La gran mayoría de los estudios de la medicina tradicional excluyen la existencia de la biomedicina y de las medicinas alternativas y complementarias en la vida de los pueblos indígenas de México y de Latinoamérica en general, pese a que estos pueblos utilizan la biomedicina en forma creciente e intensa. En este texto, he tratado de poner de manifiesto este proceso de expansión biomédica y de declive de la medicina tradicional, a través de información etnográfica referida a distintos pueblos originarios. Esta expansión biomédica se desarrolla a pesar de las varias consecuencias negativas que genera, debido a diversos factores, entre ellos, su eficacia comparativa, que se expresa a través de los usos y la demanda de fármacos, de los servicios biomédicos y, en particular, de la instalación de hospitales en sus comunidades. La población indígena articula los usos de la medicina tradicional y de la biomedicina con la tendencia a utilizar cada vez más la biomedicina, incluso por parte de los curadores tradicionales.

## Abstract (en)

The vast majority of studies on traditional medicine disregard the existence of biomedicine and alternative and complementary medicines in the lives of the indigenous peoples of Mexico and Latin America in general, despite the fact that these populations increasingly make use of biomedical knowledge more and more intensively. In this text I have attempted to elucidate this expansion of biomedicine and the decline of traditional medicine, through ethnographic information related to different indigenous groups. This expansion of biomedicine takes place despite the various negative consequences it generates due to different factors such as its comparative effectiveness, which is evidenced in the use of and demand for pharmaceuticals, biomedical services, and in particular the construction of hospitals in their communities. The indigenous population combines the uses of traditional medicine and biomedicine with a tendency to increasingly utilize biomedicine, even on the part of traditional healers.

## INTRODUCCIÓN

En México, actualmente todos los sectores sociales, incluidos los pueblos originarios, utilizan diferentes formas de atención y prevención. En términos esquemáticos, dichos pueblos utilizan autoatención, medicina tradicional, biomedicina, así como algunas medicinas alternativas y complementarias. El uso de estas formas no es un proceso reciente, pero considero que se impulsó, sobre todo, a finales del siglo XIX y, especialmente a partir de las décadas de 1930, 1940 y 1950, de tal manera que los diferentes grupos originarios utilizan en su vida cotidiana formas de atención y prevención que implican técnicas, procesos y conceptos que no solo devienen de la medicina tradicional. No cabe duda de que hay diferencias sociales, culturales y de poder en los diferentes grupos originarios mexicanos, que también operan en el caso de la medicina tradicional, pero en la medida que asumamos que las principales enfermedades tradicionales y no tradicionales, al igual que los tipos de curadores tradicionales, son similares en todos los grupos originarios^1^^,^^2^.

Pero lo que observamos en los estudios sobre medicina tradicional es la exclusión de la biomedicina y de las medicinas alternativas y complementarias, pese a que estas se caracterizan por ser operadas en forma articulada por los sujetos y grupos nativos y pese a que gran parte de los analistas reconocen su presencia en la vida de los pueblos indios, tal como fue señalado tempranamente por Menéndez^3^^,^^4^ y Zolla^5^. Esta orientación excluyente se ha mantenido como dominante, pese a dos procesos: a) la expansión constante de la biomedicina y el uso de servicios y productos médicos por parte de las comunidades indígenas, desde por lo menos la década de 1920 hasta la actualidad; y b) la emergencia de padecimientos crónico-degenerativos, especialmente cardiovasculares y diabetes mellitus 2, que los pueblos originarios resignifican en función de enfermedades tradicionales, pero incluyendo central o complementariamente la dimensión biomédica (en este texto solo analizaremos las exclusiones generadas respecto de la biomedicina). 

Dicha exclusión se desarrolla debido a diferentes objetivos. En primer lugar, porque se busca lo propio, lo intrínseco, lo que expresa la cultura de los grupos originarios, que solo estaría en la medicina tradicional. En segundo lugar, porque se ve la expansión biomédica sobre los pueblos originarios como negativa, en términos de identidad cultural, dado que tiende a aculturar, a dislocar, a impulsar la desaparición de las formas propias nativas. En tercer lugar, porque se ve a la biomedicina no solo como aculturadora, sino porque generaría consecuencias negativas en los saberes de la población indígena, dado el dominio del biologismo, del mercantilismo, del individualismo, del etnocentrismo, del racionalismo “occidental”, del colonialismo en la biomedicina^6^^,^^7^. Y, por último, porque la biomedicina no solo no soluciona ciertos problemas de salud como es el caso de las enfermedades cardiovasculares, sino que en muchos aspectos no consigue reemplazar a la medicina tradicional en la atención de padecimientos. 

Marcos Arana Cedeño, uno de los mejores conocedores de los procesos de salud-enfermedad-atención-prevención en Chiapas, considera que la expansión biomédica es muy clara cuando se trata de padecimientos agudos o de tratamientos quirúrgicos, lo que no ocurre en el caso de las enfermedades crónico/degenerativas, ya que la población encuentra en la medicina tradicional mejores respuestas que en la biomedicina, en gran medida porque los tratamientos médicos pueden ser muy largos y costosos y/o no tienen eficacia (Arana Cedeño, 2015, comunicación personal). Esto se evidenció durante la pandemia de covid-19, dado que la población vivió la inconsistencia de la información y de las actividades del sector salud, lo que alentó su desconfianza.

Sin embargo, gran parte de estas propuestas constituyen presupuestos, dado que casi no contamos con estudios generados por los estudiosos de la medicina tradicional, que describan y analicen etnográficamente dichas propuestas; mientras en nuestros estudios sobre Yucatán hemos evidenciado la constante expansión biomédica; sin olvidar que, por lo menos entre las décadas de 1960 y 1990, era el estado con mayor población indígena. Estos estudios los complementé con los realizados en otros estados (Coahuila, Guanajuaro, Michoacán, Veracruz, Zona Lagunera), así como con una extensa consulta bibliográfica y la solicitud de información a varios de los principales especialistas mexicanos en medicina tradicional. Aclaro que dicha consulta a especialistas, así como la revisión de revistas y periódicos fue debida a la escasez o directamente inexistencia de estudios sobre varios de los procesos que analizaré.

## EXPANSIÓN BIOMÉDICA: ALGUNAS CARACTERISTICAS BÁSICAS

Parto del supuesto teórico y etnográfico de que la biomedicina se caracteriza por su expansión permanente y constante sobre los diferentes sectores sociales mexicanos, incluidos los pueblos originarios, lo que podemos observar en términos antropológicos desde la década de 1920, a través de estudios realizados desde la antropología estadounidense por Redfield^8^^,^^9^, Beals^10^, Foster^11^, Holland^12^, Harman^13^, Zing^14^ y, desde la antropología mexicana, por Gamio, Caso, De la Fuente, Aguirre Beltran y otros^15^. Pese a esta expansión, ha dominado la idea de que los grupos originarios no solo prefieren más la medicina tradicional, sino que rechazan la biomedicina o ciertos aspectos de ella, lo cual es correcto parcialmente para ciertos momentos históricos (por ejemplo, entre las décadas de 1920 y 1970), pero que no corresponde a la situación actual. Las investigaciones de Guzman^16^, Menéndez^4^ y Ramírez^17^ han evidenciado el incremento de los servicios biomédicos en dos comunidades yucatecas rurales entre 1920 y 1980; incremento que no solo se observa en el caso de los servicios médicos oficiales, que suelen incluir comités de salud integrados por población nativa, sino también en el incremento constante de servicios médicos privados, de farmacias, de curadores tradicionales supervisados por el sector salud, así como de organizaciones no gubernamentales (ONG) y de grupos de Alcohólicos Anónimos^18^. 

Los estudios realizados en Ticul y Pustunich, en la década de 1970, mostraron que la carrera de la persona enferma, así como toda la autoatención evidenciaban que la población de estas comunidades utilizaba diversas formas de atención de los padecimientos, incluidas la biomedicina y la medicina tradicional. Más aun, mostraron los procesos transaccionales que la población hacía entre dichas formas de atención, que se caracterizaban por el incremento de la expansión y demanda biomédica^4^. Y este proceso ocurría pese a que las políticas públicas de salud marginaron a los pueblos indios, especialmente cuando desde la década de 1940 se impulsó un gran desarrollo de servicios médicos, los cuales fueron creados casi exclusivamente para sectores urbanos y pertenecientes a la mano de obra privada y de funcionarios públicos. Esta marginación perdura hasta la actualidad, y se expresa paradigmáticamente en palabras de un alto funcionario de la Secretaría de Salud, quien considera que el enfrentamiento a la diabetes mellitus 2 no incluye trabajar con grupos indígenas, sino con población de alto impacto (comunicación personal).

La expansión biomédica en las zonas indígenas se puede observar en el incremento de personal e instituciones médicas y paramédicas, pero también a través de datos epidemiológicos que evidencian el impacto biomédico en la reducción de la mortalidad si vemos, por ejemplo, quienes han atendido los partos entre 2000 y 2005 a nivel nacional y a nivel de los estados con mayor población indígena, observamos un fuerte incremento de los partos atendidos por la biomedicina^19^. Esta expansión biomédica, si bien es reconocida no es, como ya señalamos, descripta y analizada antropológicamente en los pueblos originarios. Más aún, se tiende a formular críticas correctas a la biomedicina como, por ejemplo, sus funciones de control social e ideológico, pero sin que la mayoría de los analistas consideren también en términos de control social e ideológico a los curadores tradicionales. Entre 2017 y 2022, cinco analistas me han señalado que desean no abordar las enfermedades y curadores tradicionales como controles sociales para no evidenciar cómo a través de dichas enfermedades y curadores se legitiman relaciones de dominación, explotación y violencia. Una reputada investigadora, cuyo nombre omito, considera que en sus estudios sobre salud reproductiva no consigna ciertos datos que revelan que las mujeres indias embarazadas prefieren a la médica o al médico y no a la partera “porque me van a decir que ahora me medicalicé, que ya no defiendo a las parteras. Y aunque no me lo digan, siento que estoy traicionando a las parteras si obtengo ciertos datos”.

Correlativamente, observamos que al igual que respecto del control se habla de “mala práctica” médica, no se habla y menos se analiza la “mala práctica” de los curadores tradicionales, aunque tampoco se estudia “mala práctica” médica, salvo en lo relativo a violencia obstétrica. La mayoría de los textos, en forma explícita o larvada, critican a la biomedicina y a la “racionalidad occidental”, pero a partir de supuestos ideológicos, dado que no describen ni analizan la biomedicina, y menos aún la “racionalidad occidental”. Analizan además los procesos de salud-enfermedad-atención-prevención, como si la medicina tradicional implicara cosmovisiones y cultura, mientras la biomedicina y los “occidentales” no.

Si bien la expansión biomédica sobre las áreas rurales y las indígenas, en particular, se inicia y desarrolla previamente, observamos que desde la década de 1920 se va a generar toda una serie de actividades, varias transitorias y otras constantes, que implican a un sector salud que se caracteriza por su hegemonía y dominio sobre el campo de la salud-enfermedad-atención-prevención^20^.

Dicha expansión se realiza dentro de procesos que cuestionan aspectos de la biomedicina, mientras otros impulsan su demanda. Las principales críticas que se hacen a la atención médica podemos organizarlas en varios apartados:

1. Las que remiten al trato vertical, impersonal y autoritario; al mal trato al paciente; a que no entienden a la población; el personal de salud y sobre todo las médicas y los médicos no hablan la lengua de sus pacientes; tratan a las personas como ignorantes, irracionales, inferiores; al largo tiempo de espera para la consulta; al horario restringido; a la escasez de personal, a su rotación y a su ausentismo; a que atienden pasantes y no profesionales titulados; a que el personal médico no está capacitado; a que los medicamentos que dan en los centros de salud son menos eficaces que los recetados por la medicina privada; a la falta de medicamentos.
2. El personal de salud no reconoce y desvaloriza la medicina tradicional; pero correlativamente observamos que una parte de los curadores tradicionales cree que el personal de salud biomédico le va a robar su saber sobre los procesos de salud-enfermedad-atención-prevención.
3. A la presión ejercida sobre las mujeres para que apliquen técnicas anticonceptivas, especialmente la esterilización; a que las mujeres a punto de parir no son tratadas inmediatamente, con consecuencias graves para ellas y su “producto”; a la imposición de un parto horizontal.
4. No van a los servicios médicos por razones económicas; por el alto costo de los medicamentos.
5. La biomedicina no cura determinados padecimientos que sí curan los curadores tradicionales; el sector salud habla demasiado de prevención, cuando a la gente lo que le interesa es la atención y sanación.

Los procesos culturales, la cosmovisión, el rechazo a la biomedicina y su incompatibilidad con la medicina tradicional no aparecen en la práctica como factores que conducen al rechazo de la biomedicina. Como señala Quatrochi en la detección y tratamiento del cáncer cérvico uterino en Yucatán:

En conclusión, los elementos que alejan a las mujeres de los servicios de detección temprana de cáncer cérvicouterino no parece estar relacionados a mitos o a costumbres “arcaicas” que provienen de su cultura, como a veces de manera muy superficial, en el discurso oficial se afirma (la cultura como factor de riesgo). En realidad, hay más mitos y creencias en la literatura médica o en las opiniones institucionales; por ejemplo, hay una opinión bastante compartida de que las mujeres no acuden a los servicios de detección o no siguen el tratamiento por miedo a la enfermedad o por un inexplicable sentido de fatalidad. Casi nunca se ha encontrado esta actitud; más bien el miedo que las mujeres y las familias expresan está relacionado a la preocupación de no tener dinero para curarse, por no tener atención de calidad, por no tener un trato adecuado por parte del personal de salud.^21^

Concordando con esta autora, cabe señalar que lo propuesto no solo refiere al personal de salud sino también a la “cosmovisión” de gran parte de las antropólogas y los antropólogos, y no solo de América, dado que en gran parte de este grupo domina una visión esencialista y focalizada en la cosmovisión de los pueblos originarios. Esto lo ha señalado Andrés Cuyul para Chile a través de varios trabajos^22^^,^^23^; pero el caso más llamativo es el comentado por Jacques Lizot^24^ para los yanomami venezolanos sobre la compatibilidad entre chamanismo y biomedicina. 

Lo señalado previamente debe ser relacionado con el hecho reiterado de que por lo menos una parte de las comunidades indígenas demandan no solo la instalación de centros de salud, sino sobre todo de hospitales, dado que consideran que los especialistas pueden solucionar problemas de salud graves. Más aún, por lo menos ciertos grupos originarios distinguen entre pasantes y médicos, así como entre médicos de servicios oficiales y privados, y entre médicos generales y especialistas. Considerando que los mejores médicos son los privados y especialistas. Y estas demandas las observamos en la mayoría de los pueblos originarios de los que tenemos información, desde los rarámuris hasta los mayas yucatecos y los zapotecos oaxaqueños, pasando por los wixaritari (huicholes).

Por supuesto que la relación entre crítica, rechazo y demanda de la biomedicina por parte de los pueblos indios opera dentro de un proceso histórico que va desde el dominio de la crítica y rechazo a un uso cada vez mayor de la biomedicina, hasta convertirse en la forma de sanación más estimada. Este proceso, además, tiene que ver con que, como ya señalamos, los pueblos originarios eran y siguen siendo los que contaban con menos servicios de salud, por lo que no podían usar lo que no existía como parte de sus formas de atención. Esto se articula con el hecho de que al personal de salud y, especialmente, a las médicas y los médicos no les interesa trabajar en medios rurales. Y son estos y otros procesos los que también han conducido a que la población indígena coloque cada vez más la demanda de biomedicina en su eficacia, pasando a segundo nivel las formas de atención que consideran negativas.

La población indígena ve en los fármacos y en las inyecciones de fármacos el recurso más eficaz de la biomedicina, y por eso el uso de medicamentos constituye la principal demanda de la consulta biomédica. Una consulta médica sin receta de medicamentos es casi ininteligible para los pacientes. Además, en las comunidades hay cada vez más farmacias, pero recordando que una parte de los medicamentos se venden también en diferentes tipos de tiendas. Los medicamentos biomédicos no solo son empleados para tratar enfermedades alopáticas, sino cada vez más para tratar enfermedades tradicionales en forma articulada con la medicina tradicional. Frecuentemente, el paciente -y no solo indígena- no entiende lo que le dice el médico o la médica, pero concurre a la atención médica en busca de recetas de medicamentos para su padecimiento. Más aún, dicho uso tiene que ver, además, con el efecto inmediato “curativo” y no preventivo, como describe Cortez^25^ respecto de familias indígenas, dado que por más que hayan recibido información no utilizan los sobres de rehidratación oral. Esto también ocurre en otros contextos, como en el caso de la población hindú, que considera que la rehidratación oral no sirve para las diarreas de los niños y solicitan antibióticos o sueros intravenosos que son los que curan^26^.

Como ya señalamos, los medicamentos que compran en las farmacias recetados por médicos privados son considerados mejores que los que les dan o venden en los servicios médicos oficiales, por lo que la inversión en medicinas se convierte en un importante rubro del presupuesto familiar. Ahora bien, este proceso es común a todos los pueblos originarios de América Latina y, en el caso de Brasil, Diehl señala:

Las investigaciones sobre medicamentos en contextos locales indígenas brasileños muestran que la atención a la salud está caracterizada por la hipermedicalización que debe ser explicada no solo como un acto aislado de búsqueda de cuidado, sino como parte de un proceso que incluye a los sujetos y grupos y a los diferentes especialistas.^27^

Y en este proceso no solo interviene el sector salud y la medicina privada, sino también la industria químico-farmacéutica e instituciones internacionales de salud como Unicef, quien en 1988-1989 entregó $20.000 dólares para comprar medicamentos para grupos étnicos del estado de Oaxaca. Y es esta hipermedicalización la que conduce a que Freyermuth Enciso concluya que:

La comercialización de los productos farmacéuticos en las zonas indígenas sin el control de la Secretaría de Salud, propicia la autoamedicación o la “prescripción” sin el conocimiento de las contraindicaciones o efectos secundarios (y se hace) al margen del sistema médico nacional, lo que ha permitido a la industria farmacéutica inundar el mercado sin que la población cuente con la información mínima confiable para su uso.^28^

Ahora bien, no cabe duda de que este consumo tiene que ver con la expansión médica de instituciones de salud internacionales, de ONG y con la industria químico-farmacéutica ¿Pero la población tiene algo que ver con dicho consumo o solo actúa por influencia de fuerzas “externas”? Considero que dicho consumo no podemos atribuirlo solo a la influencia de las instituciones señaladas o a la alienación o ignorancia de la población, sino también a las funciones que cumple este consumo a partir de las condiciones de vida de la población indígena y no indígena. Necesitamos asumir, una vez más, que la mayoría de las acciones de salud en los sujetos y microgrupos se da a traves del proceso de autoatención, y el uso de fármacos se va convirtiendo en el principal medio de autoatención por varias razones que ahora no vamos a analizar. En nuestro estudio sobre el estado de Yucatán, en la comunidad de Ticul, observamos que la población tomaba refrescos en particular Coca-Cola y Delaware cuando tenía diarrea, lo cual había sido recomendado por los médicos, dado que los refrescos eran el “agua” más potable en una comunidad que carecía de agua potable. Es por eso que desde hace años considero que necesitamos generar investigaciones sobre cuáles son las reales causas y consecuencias de los procesos de autoatención, incluida la automedicación; necesitamos describir y analizar la racionalidad económica, cultural y de salud que lleva a los sujetos y microgrupos a consumir biomedicina, cuestionando que unos remitan lo que ocurre a la alineación, mientras otros niegan lo que ocurre adheridos a un escencialismo proindígena.

El uso de fármacos biomédicos no solo se da en los sujetos y familias indígenas, sino que son cada vez más utilizados por los antiguos y nuevos curadores tradicionales. De tal manera que las parteras, los hueseros, los culebreros, los yerbateros y hasta los chamanes utilizan fármacos de patente, incluido el uso de medicamentos psiquiátricos. Además, una parte de los curadores tradicionales adopta el lenguaje médico, así como las características del medio donde atienden^17^^,^^29^.

Y por eso la biomedicina en forma directa (atención médica) como indirecta (uso de fármacos por autoatención) se ha ido convirtiendo en el principal recurso para atender los padecimientos. Más aún, los procesos biomédicos analizados nos indican que el uso de fármacos se ha ido convirtiendo en parte intrínseca de la vida de los pueblos originarios, siendo parte de la medicina tradicional y del trabajo de los curadores tradicionales. Y pese a ello, estos procesos son excluidos en la mayoría de los estudios sobre la medicina tradicional de los pueblos originarios.

Pero la expansión biomédica opera básicamente a través de la atención clínica y, muy en segundo lugar, de la prevención sobre comunidades originarias que utilizan gran cantidad de criterios y actividades preventivas respecto de las enfermedades tradicionales, las violencias y otros riesgos. Esto es negado por antropólogos como Foster al considerar que la población rural no tiene noción de que hay que cuidar la salud, el cuerpo y prevenirse de enfermedades. Pero:

Una hipótesis alternativa propone que no es que falte la medicina preventiva en las zonas rurales de América Latina, sino que los administradores de salud pública con entrenamiento occidental no llegan a percibir con frecuencia las prácticas preventivas como tales, por la suposición etnocéntrica de que la enfermedad siempre tiene un origen físico [sin embargo] los datos que recopilan los antropólogos en las comunidades rurales de América Latina demuestran terminantemente que, basada en la conducta social, existe siempre una gran preocupación por la prevención de las enfermedades, y que los miembros de cada grupo comparten medidas preventivas bien definidas.^13^

La notable presencia de actividades preventivas para todas las enfermedades tradicionales, para el embarazo-parto, para violencias y para procesos ambientales la hemos encontrado en comunidades de Yucatán, juntamente con la constante consulta con los diferentes curadores tradicionales, pero observando que la población nativa reconoce y demanda básicamente atención en el caso de la biomedicina.

## ARTICULACIONES ENTRE MEDICINA TRADICIONAL Y BIOMEDICINA

Desde la década de 1920 tenemos información etnográfica acerca de que la carrera del enfermo se inicia con la autoatención pero, de inmediato, si no da resultado, implica la consulta inicial al curador tradicional y, si no hay sanación, se pide una consulta biomédica. Actualmente, la primera acción sigue siendo la autoatención mientras, para enfermedades graves físicas, se demanda consulta biomédica. Erasmus presenta datos para México a partir de 1910, y señala que entonces dominaba totalmente la medicina tradicional, pero

Hoy hasta habitantes de una comunidad bastante conservadora y con predominio indio como Mezquital suelen confiarse al cuidado de un médico moderno […] Se pidió a los jefes de 42 familias de Mezquital que describieran el curso del tratamiento de las enfermedades graves sufridas más recientemente por algún miembro de sus familias. Si bien en el 74% de los casos el tratamiento comenzaba con remedios caseros, solo lo proseguían hasta el final el 7%. En un 18% de los casos el tratamiento fue iniciado por un curandero y en un 23% se lo consultó como paso intermedio. El tratamiento concluyó en manos de un curandero solo en un 10%. El farmacéutico fue paso intermedio en el 7% y final en el 21%. Los médicos fueron paso inicial en un 7% y concluyeron el tratamiento en un 62% de los casos.^30^

La articulación autoatención-medicina tradicional-biomedicina observada en el tiempo implica el paso a primer plano y como forma dominante de la biomedicina, lo que sobre todo se observa a travéz de su impacto en el proceso de embarazo y parto que, de estar casi totalmente en manos de parteras “empíricas” hasta mediados de la década de 1970, pasó a ser casi exclusivamente realizado por biomédicos^31^^,^^32^.

En este proceso, es importante recuperar que las relaciones entre las diferentes formas de atención, y sobre todo entre la medicina tradicional y la biomedicina, fueron replanteadas desde la década de 1980 en términos de interculturalidad. Si bien la interculturalidad se había propuesto desde la década de 1940, en términos de aculturación, durante el neoliberalismo va a ser propuesta en términos de reconocimientos simétricos culturales, utilizando los conceptos de interculturalismo y de multiculturalismo para subrayar, sobre todo, en el segundo caso, el peso de lo cultural nativo. Sin embargo, en el caso de México -como en otros países latinoamericanos- ambos conceptos fueron utilizados buscando objetivos similares^33^. Los interculturalistas plantean un “dialogo de saberes”, una armonía entre los actores sociales en relación, una relación en la que lo cultural aparece como prioritario y excluyente. Se propone una interculturalidad basada en relaciones de respeto y complementarias entre actores e instituciones pertenecientes a culturas diferentes; en el reconocimiento del otro y el trato igualitario en el cual se reconozcan y acepten las diferencias en lugar de cuestionarlas. Y así, para Walsh, la interculturalidad significa:

…un intercambio que se establece (entre actores culturales) en términos equitativos, en condiciones de igualdad […] la interculturalidad debería ser entendida como un proceso permanente de relación, comunicación y aprendizaje entre personas, grupos, conocimientos, valores y tradiciones distintas, orientadas a generar, construir y propiciar un respeto mutuo, y a un desarrollo pleno de la capacidad de los individuos, por encima de sus diferencias culturales y sociales.^33^

Si bien se reconocen varios tipos de interculturalidad, en algunas de las cuales se plantean los conflictos o desigualdades como procesos a tomar en cuenta, los usos del sector salud y de las instituciones antropológicas oficiales remiten casi exclusivamente a relaciones culturales sin conflicto o con conflictos superables, mientras corrientes indigenistas generalmente esencialistas proponen una incompatibilidad radical.

En términos concretos y específicos, la recuperación de las relaciones interculturales tiene que ver con las relaciones que pueden establecerse entre biomedicina y medicina tradicional en términos complementarios, para extender la consulta y mejorar la atención primaria de la salud-enfermedad. Esto condujo a que, desde mediados de la década de 1970, se impulsen programas de salud que van a incluir curadores tradicionales, pese a que el personal y especialmente los médicos los cuestionan y rechazan. Dicha propuesta es generada desde el sector salud y complementada con propuestas que vienen del Instituto Nacional Indigenista, y se expresa a través de dos actividades básicas: la creación de hospitales, clínicas o centros de salud mixtos, en los que operan en forma conjunta pero separada el personal biomédico y los curadores tradicionales. Y, en segundo lugar, la creación de organizaciones de curadores tradicionales para legitimar social, cultural y legalmente su ejercicio sanador. Ambos tipos de actividades, salvo excepción, fueron generadas, impulsadas y financiadas por instituciones del Estado o extranjeras y no por las comunidades indígenas. El mayor y permanente financiamiento respecto de las organizaciones de curadores tradicionales fue realizado por el Instituto Nacional Indigenista. En el caso de algunas de las pocas organizaciones indígenas que siguen funcionando, observamos que las parteras empíricas de Chiapas tuvieron financiamiento del Instituto Nacional Indigenista, de Unicef y de la Secretaría de Salud, mientras que la Organización de Médicos Indígenas del Estado de Chiapas, lo obtuvo de la Fundación Pan para el Mundo, Fundación Chagas y de la embajada de Holanda en México. De 1989 a 1995, el financiamiento de estas organizaciones de curadores tradicionales que funcionan en Chiapas fue realizado por instituciones del Estado.

Ahora bien, los dos tipos de actividades señaladas fracasaron de tal manera que la mayoría de las organizaciones de curadores tradicionales han desaparecido o languidecido, y los hospitales mixtos se caracterizan por su escaso uso o por un uso turístico, así como por la hegemonía biomédica respecto de la medicina tradicional en todos los planos de la realidad^25^^,^^34^^,^^35^^,^^36^^,^^37^^,^^38^^,^^39^^,^^40^.

En el caso de los procesos impulsados por el sector salud, en la práctica se dio un uso subordinado de los curadores tradicionales buscando objetivos de salud específicos y, especialmente, la reducción de la tasa de natalidad. En la práctica, no se buscó respeto a la diferencia, armonía y complementación no subordinada entre los actores, por lo que el sector salud cumplió con sus objetivos biomédicos reales y no con las propuestas discursivas proindígenas. En el caso de las actividades interculturales impulsadas por el Instituto Nacional Indigenista se propuso una interculturalidad basada en el respeto, la armonía, el reconocimiento del otro, etc., sin embargo, lo hizo solo a partir de las características culturales de la relación, excluyendo los procesos de desigualdades sociales, económicos, de poder, la situación de pobreza y extrema pobreza y de marginación indígena^41^^,^^42^, sin tomar en cuenta los procesos de hegemonía-subalternidad-contrahegemonía existentes. Si bien algunos interculturalistas reconocen la importancia de estos procesos, no son incluidos en sus análisis y menos en sus intervenciones.

El Instituto Nacional Indigenista ignoró en la práctica las relaciones etnocéntricas, racistas y clasistas del personal de salud respecto de los grupos subalternos y, sobre todo, no asumió ni teórica ni prácticamente que las relaciones sociales actuales están basadas en las relaciones previas que tuvieron los pueblos originarios con la biomedicina, y que están caracterizados por la violencia estructural.

Y este fracaso lo reconocen algunos de los principales especialistas en interculturalidad como Walsh^33^, dado que si bien se han hecho cambios legislativos importantes respecto de la interculturalidad, sin embargo, en la práctica, todo sigue igual. Así es como Fernández Juárez -posiblemente el mayor impulsor de la publicación de textos sobre interculturalidad en Iberoamérica- considera que la interculturalidad

*“…fracasó con mayúscula, se perdió hace tiempo el centro, el eje, la columna que debía haber sido el enfermo, el ciudadano, y se adornó en exceso el “escaparate político” con peso inasumible en lo cultural. Por eso, si solo se trata de “cosas de indios” o de “inmigrantes”, mejor dejar. Hay que criticar el modelo y no seguir ordeñando a las vacas sin sentido*”. (Fernández Juárez, Comunicación personal, 2015)

Ahora bien, la articulación entre biomedicina y medicina tradicional apareció legitimada e impulsada, como sabemos, a partir de la conferencia de Alma Ata de la Organización Mundial de la Salud (OMS) en 1978, que propuso lograr la cobertura total de salud a nivel mundial para el año 2000, incorporando la medicina tradicional. Esta propuesta fue asumida por la Organización Panamericana de la Salud (OPS) en la década de 1980 y, sobre todo, en la de 1990, al plantear en 1992 la necesidad de comenzar a hacer consultas para desarrollar el trabajo en salud con los pueblos indígenas, y en 1993 propuso el desarrollo de un enfoque integral en salud, reconociendo el derecho a la autodeterminación de los pueblos originarios, así como el respecto a las culturas indígenas y una relación de reciprocidad con ellas. En 1998, como síntesis de sus propuestas, reconocen las capacidades de la medicina tradicional para enfrentar los problemas de salud de sus comunidades^43^^,^^44^, asumiendo la capacidad y necesidad de trabajar con los curadores tradicionales y, por lo tanto, incluir los saberes indígenas en las actividades de salud:

A través del diálogo horizontal, la interculturalidad aspira al reconocimiento y valoración de conocimientos y prácticas de salud locales -así como a la incorporación de las mismas dentro de los sistemas de salud convencionales- como una herramienta no solo para la aceptabilidad de los sistemas de salud y para la consolidación de un sistema más equitativo y participativo, sino para lograr, además, un mundo más justo y humano.^45^

La OPS propuso un plan de acción y metas en salud para el Decenio Internacional de los Poblaciones Indígenas del Mundo (1995-2004), que no se cumplieron. Más aún, manejaba una concepción de la medicina tradicional en términos esencialistas, que colocaba el eje en la cosmovisión^43^. Sin embargo, en lugar de impulsar una atención primaria integral acorde con estos planteamientos, desarrolla una atención primaria focal y selectiva que centraliza todo en la biomedicina, lo que se relaciona con el hecho de que a los médicos no les interesa el enfoque intercultural.

Esta tendencia, aparentemente contradictoria de la OPS, se observa en la trayectoria de los gobiernos mexicanos entre 1980 y 2023 respecto de la medicina tradicional, dado que se promulga y/o se verbalizan toda una serie de propuestas que impulsarían la medicina tradicional, pero que realmente solo la utilizan para luego excluirlas y/o subordinarlas. Los gobiernos neoliberales impulsaron el reconocimiento y uso de la medicina tradicional desde que Salinas de Gortari fue candidato a presidente de la República en la década de 1980 y en su campaña para obtener la presidencia sostuvo:

Hay que dar pasos hacia adelante en la atención primaria a la salud y fundamentalmente en la prevención, tomando en cuenta los métodos tradicionales propios de cada comunidad. El método es una opción adecuada para atender la demanda insatisfecha en el medio rural y para contribuir a la organización social, que es parte del bienestar en el campo.^46^

Sin embargo, durante el sexenio del presidente Salinas de Gortari, mientras se reconoce la importancia de la medicina tradicional, en 1992 se reforma el Artículo 27 de la Constitución para liberar la venta de tierras comunales y ejidatarias que, en gran parte, están en territorios indígenas.

Las reformas constitucionales mexicanas de 1990 y 2001 no solo reconocen a México como nación pluricultural sustentada en sus pueblos indígenas, sino que reconocen la existencia y papel de la medicina tradicional; pero dichas reformas no se tradujeron en modificaciones en la ley de salud respecto de la medicina tradicional. 

Durante la primera década del 2000, se crean las universidades interculturales -varias de las cuales tienen escuelas de salud- y se reconoce el estatus de las lenguas indígena en equivalencia con el español, y la obligación de las instituciones de salud de tener personas que operen como traductores bilingües. La Secretaría de Salud crea la Comisión de Salud para los pueblos indígenas, y el gobierno crea la Comisión Nacional para el Desarrollo de los Pueblos Indígenas^47^, proponiendo acciones articuladas con el sector salud. Correlativamente, los estados de Chiapas, Ciudad de México, Morelos, Oaxaca, Queretaro, Nayarit y Veracruz reconocen el papel de la medicina tradicional. Incluso en el estado de Veracruz la Secretaría de Salud, en 2013 y 2014, desarrolló un modelo de salud indígena con pertinencia cultural, pero que luego desaparece por razones políticas^48^. Esto se reitera en el Programa Nacional de Salud para el período 2007-2011, en el que se plantea incorporar gradualmente servicios de medicina tradicional en las unidades de salud que exista demanda de medicina tradicional, lo que se generó en forma anecdótica.

La política de salud, desarollada desde mediados de la década de 1970, está basada en propuestas de la OMS de utilizar la medicina tradicional como recurso de atención y prevención, que se fundamenta en las concepciones de McKeown, quien considera que la atención médica no es lo relevante, sino que son determinantes las condiciones de vida incluida la alimentación^15^. Pero esta orientación aparece en el discurso del sector salud, pero no en la práctica, dado que siguen basadas en la atención biomédica. Sin embargo, pese a que a nivel constitucional y de la ley general de salud se reconoce y se promueve la medicina tradicional en México, y pese a que la mayoría de las constituciones y leyes estatales a principios del siglo XXI han generado modificaciones que reconocen la medicina tradicional, “la situación de la medicina indígena se encuentra estancada pues no existe un programa nacional especifico, y los apoyos políticos, sociales y financieros brillan por su ausencia”^35^^,^^49^^,^^50^^,^^51^.

Pero además, de los intercambios con especialistas como Barragán, Campos, Güémez, Haro, López, Maya, Mendoza, Page, realizados entre 2018 y 2023, queda claro que este impulso discursivo contrasta con la fuerte reducción del número de curadores tradicionales, con su biomedicalización y con la exclusión de curadores tradicionales que se dedican a la brujería, a la adivinación y similares^4^^,^^17^^,^^37^^,^^40^^,^^52^^,^^53^^,^^54^^,^^55^. Un importante especialista como Armando Haro, si bien reconoce la disminución del número de curadores tradicionales en los pueblos originarios de Sonora, señala que eso no quiere decir que la medicina tradicional está desapareciendo (comunicación personal, 2021), lo cual es correcto, pero una de las cuestiones a analizar reside en que los curadores son los que no solo realizan actividades sanadoras y/o enfermantes, sino los que manejan las cosmovisiones que sostienen sus saberes, siendo importante reconocer que no tenemos investigaciones sobre el proceso de biomedicalización de los curadores tradicionales, ni tampoco sobre la “medicalización” que ejercen los curadores tradicionales. 

Es necesario asumir el alto número de curadores tradicionales que operan en las comunidades indígenas. Por ejemplo, Metzger y Williams^56^ señalan que, en Tenejapa, en una población de alrededor de 10.000 personas, la relación de curandero por habitante es 1 a 19 o de 1 a 25 habitantes. Lummholtz^57^, a principios de siglos XX, encontró que la relación entre los huicholes era de 1 a 7 habitantes. En 1993, Freyermuth Enciso estimó que en los altos de Chiapas había una relación de un curandero por 120 habitantes, mientras que entre médicos/promotores de salud y población había una relación de 1 a 1.000 habitantes^53^.

Pero el caso paradigmático ya mencionado es el de las parteras empíricas, dado que eran quienes realizaban la mayoría de los partos en las zonas indígenas, y fue el recurso tradicional más utilizado por el sector salud, especialmente desde mediados de la década de 1970, posibilitando en un inicio que realizaran partos, para ir limitando cada vez más sus acciones hasta quedar excluidas en la actualidad de la realización de partos, y operando como educadoras de la salud, sobre todo respecto de la anticoncepción y como sobadoras. Como ya señalamos, esto tuvo que ver con la aplicación del Programa de Planificación Familiar, que se convirtió en el principal programa de atención primaria^17^^,^^25^^,^^31^^,^^32^^,^^35^^,^^49^^,^^58^^,^^59^^,^^60^. Este proceso de medicalización y exclusión de las parteras empíricas, si bien ha tenido una gran continuidad, hubo intermitencias, como la generada por la eliminación del Seguro Popular en 2018. En los tres años posteriores, al menos en ciertas zonas indígenas resurgió el trabajo de las parteras al desaparecer o disminuir la oferta de servicios biomédicos^32^. Más aún, si bien el Instituto de Salud para el Bienestar, que reemplazo al Seguro Popular, propuso contar con el trabajo de las parteras empíricas, ello no se dio en la práctica y condujo a que estas, entre 2018 y 2022, exigieran no solo reconocimiento, sino la permisión de su trabajo como parteras, ya que denuncian que han sido excluidas de la realización de partos^61^^,^^62^.

Este proceso, con diferentes gestiones, se ha dado en toda América Latina. En él se busca realmente la aculturación/asimilación de los pueblos originarios a travéz de la expansión biomédica, pero no como un objetivo en sí, sino como consecuencia de dicha expansión^63^. En un texto en que Ramírez^64^ analiza la interculturalidad en Bolivia, señaló que esta tiene como objetivo central apaciguar y controlar a los sectores subalternos, concluyendo que en Bolivia y también en Ecuador no funcionan los servicios de salud intercultural. 

## INTERPRETACIONES POSIBLES

Del material analizado surge que la biomedicina no solo se expande, sino que va pasando a ser la forma más utilizada luego de la autoatención, que opera una carrera de la persona enferma que incluye las tres formas básicas ya señaladas, pero con hegemonía biomédica. Se observa también que disminuye cada vez más el número de curadores tradicionales, algunos en forma radical, como en el caso de los j’men de Yucatan que pasaron de 400 a ser solo alrededor de 20. Los datos presentados indican no solo una expansión constante de los servicios médicos, sino la aplicación de programas de atención primaria como el de Planificación Familiar que va a imponer -y más tarde consensuar- el uso de diferentes tipos de anticonceptivos, incluyendo la esterilización y las cesáreas. Y si bien los servicios médicos que se expanden en áreas indígenas son de menor calidad y diversidad, se incrementa su presencia, que es lo que registra parte de la población indígena.

Hay varios aspectos presentados que indican con claridad no solo la expansión biomédica, sino el papel de las relaciones de hegemonía/subalternidad, como son el hecho de que la atención biomédica y especialmente la especializada y la que hay que pagar sean las mejores consideradas por la población indígena, por lo menos de algunas regiones. Y no solo en México, sino a nivel latinoamericano. Así, por ejemplo, en comunidades aymaras de Bolivia, la población considera que “la medicina gratuita del hospital no es buena, y como no se paga, se recibe mala atención”^65^, de forma similar a lo que nosotros encontramos entre 1977 y 1980 en cinco comunidades de Yucatán.

El Instituto Nacional Indigenista, en función de la aplicación del plan de desarrollo 2001-2006, realizó una encuesta a población indígena de 23 estados del país, para detectar cuáles eran sus principales demandas, y entre ellas aparecieron protagónicamente las de salud. Las personas participantes destacaron la necesidad de contar con más y mejores unidades médicas equipadas con rayos X, oxígeno, ambulancia, medicamentos suficientes y materiales de curación y, al mismo tiempo, solicitaron la legalización de la medicina tradicional. Cabe señalar que cada vez más los pueblos indígenas reclaman la localización de servicios de salud y, especialmente, de hospitales, como queda ejemplificado en el hecho de que los ocotepanos de Chiapas “piden a gritos que se concluya el hospital de Carrizal”^25^. Este proceso de demanda biomédica se ha acelerado en las generaciones más jóvenes de indígenas de Chiapas, Chihuahua, Guerrero, Oaxaca, Sonora y Yucatán, que no solo demandan servicios médicos, sino que, por ejemplo, ya no les interesa trabajar como curadores tradicionales. Si bien no tenemos información para México, observamos que en zonas indígenas de algunos países se han desarrollado cultos de médicos, especialmente por sectores subalternos^66^^,^^67^.

La población, como ya se señaló, va adquiriendo y usando cada vez más un lenguaje biomédico; utilizan los términos infección, contagio, inyecciones, medicinas de patente, riesgo obstétrico, parásitos, depresión. Ya saben que existen virus y bacterias, pero también pueden manejar concepciones eugenésicas, aunque no lo sepan ni los sujetos ni los médicos. En México, a nivel popular, el labio leporino y el paladar hundido tienen que ver con un padre alcohólico, lo que aparece corroborado en algunas etnografías como la de Castaldo, quien señala que en una comunidad nahuatl de Puebla, los niños pueden nacer mentalmente trastornados si el padre tiene relaciones sexuales con la madre mientras está borracho. Por lo que es muy frecuente que nazcan niños mutilados, y según la población “antes pensábamos que si un niño nacía sin pies o brazos era que la luna se los comía”^68^. Lo que ahora -sin saberlo- refiere a las concepciones eugenésicas desarrolladas desde finales del siglo XIX y expandidas en México por el sector médico y el educativo.

El susto se atiende, cada vez más, en hospitales, aunque en términos de enfermedades crónico-degenerativas; pero, además, la gente -según la población- muere ahora de enfermedades alopáticas, y muere cada vez menos de enfermedades tradicionales. En todo este proceso, el uso del fármacos tiene un papel decisivo, dado que emerge como uno de los principales símbolos de la eficacia biomédica, y no solo en México: “actualmente -en grupos indígenas brasileños- es bastante frecuente que el medicamento de patente sea lo primero que se use, incluso para enfermedades tradicionales”^69^.

Surge también que mientras los médicos en general rechazan toda relación con los curadores tradicionales, las autoridades del sector salud han impulsado un uso pragmático de dichos curadores y, especialmente, de las parteras empíricas, según el cual utilizan a los curadores en la medida que aseguren la realización de los programas biomédicos, no solo en forma subordinada, sino excluyéndolos cuando ya no los consideran necesarios. Pero esta exclusión, por ejemplo, en el caso de las parteras empíricas, da cuenta de los objetivos y de la racionalidad biomédica y del sector salud, pero ¿cuál es la racionalidad y las actitudes de la población?, dado que la partera empírica no solo cumplía funciones de sanación durante el embarazo y parto, sino que realizaba frecuentemente el parto en la casa de la paciente y, además, tenía un importante papel de ayuda familiar durante el puerperio (hacer la comida, lavar la ropa, asear la casa, atender al niño o a la niña). Pero pese a estas funciones, la partera fue siendo desplazada y eliminada, con muy escaso nivel de oposición por parte de las mujeres, de su grupo familiar y de las parteras.

Y esto lo logra una biomedicina que no dialoga, que no respeta los saberes tradicionales; una biomedicina vertical y autoritaria que impone un parto horizontal en lugar del parto en cuclillas; que impone esterilizaciones y cesáreas con presiones sociales, profesionales y psicológicas, que se ejercen básicamente sobre la mujer, una mujer que aparece subordinada en términos de género, sociales y raciales. Pero esto implica también la “aceptación” del varón, por lo que la biomedicina no solo se impone a la mujer, sino también a su pareja masculina y a sus familias. Y no conocemos procesos, pero tampoco estudios, que den cuenta de movimientos de mujeres, de varones y de sus familias para oponerse al tratamiento biomédico del parto y, especialmente, a la aplicación de técnicas anticonceptivas. Lo que sí tenemos son investigaciones que evidencian la expansión biomédica en términos de imposición y/o de hegemonía, como ocurre con la expansión del uso de anticonceptivos, dado que el 75% de las mujeres indígenas conocen métodos anticonceptivos y un 40% de la población indígena utiliza métodos definitivos^41^.

El personal de salud y, en particular, los médicos generan -como ya señalamos- una relación médico-paciente en la cual inferiorizan/estigmatizan/excluyen los saberes tradicionales, señalando su ineficacia, su falta de cientificidad, su irracionalidad. El personal de salud y, especialmente, los médicos y las médicas, no tienen ningún interés en incluir o articular con la medicina tradicional; pero ocurre que varios médicos que sí recuperan el papel y las funciones de la medicina tradicional a nivel discursivo, en la práctica contribuyen a excluirla. A partir de 2010, se desarrolló el programa de medicina integrativa en el Distrito Federal “en el que se incluye la homeopatía, herbolaria, quiropracia, acupuntura y naturoterapia, entre otros, que tiene como propósito su integración en las unidades de atención”^70^. El equipo estuvo formado, inicialmente, por siete médicos especializados en fitoterapia, cuatro en homeopatía y dos en acupuntrura, y pese a que el trabajo con plantas medicinales constituye uno de los principales ejes, no se incluyen curadores tradicionales. Subrayando que algunas de las personas del equipo han sido quienes más han propuesto la inclusión de curadores tradicionales en el sector salud. Según María Eugenia Modena, en 2021, en el hospital de Topilejo, donde funciona este programa, un 85% de la consulta fue alopática y un 15% correspondió a medicinas alternativas y complementarias (comunicación personal). Es interesante señalar que, en el Distrito Federal, en 2017, funcionan 40 Casas de Salud en las que trabajaban curadores tradicionales, pero dichas Casas no operan dentro del sector salud. 

Un factor importante en la demanda de atención biomédica radica no tanto en la eficacia de la biomedicina, sino en una mayor eficacia que la medicina tradicional, lo que es reconocido por la población indígena que considera que la reducción de la mortalidad general y de la mortalidad infantil, en particular, se debe en gran medida a la biomedicina. Como señalamos, uno de los sesgos de los estudios sobre medicina tradicional es no describir ni analizar etnográficamente el trabajo de los curadores tradicionales y, especialmente, de las parteras, en relación con la mortalidad de sus pacientes. No sabemos nada al respecto, y mientras se estudia la violencia obstétrica biomédica^71^, no tenemos estudios sobre la violencia obstétrica en los estudios de salud reproductiva tradicional. 

Una pregunta básica respecto de lo analizado es la que plantea ¿qué hace la población indígena y los curadores tradicionales frente a la expansión biomédica que la está desplazando, subordinando, medicalizándola? Respecto de esto, encontramos autores como Argueta *et al*.^72^, que enumeran toda una serie de reconocimientos de la medicina tradicional por el estado mexicano, y concluyen:

Como puede apreciarse, del recuento sucinto de declaraciones internacionales y regionales de 1978 a 2009 ha habido un avance enorme en los organismos regionales y mundiales de salud (OPS y OMS) en apoyo del desarrollo de la medicina tradicional [que] ha ido ganando terreno […] pero sin lugar a dudas el mayor impulso proviene de los planteamientos y las demandas de los propios médicos indígenas tradicionales, aunado a la lucha política y social de los pueblos y organizaciones indígenas del país.^72^

Cabe recordar que algunos de estos aspectos habían sido sostenidos por Favre quince años antes, al proponer que el Estado neoliberal ha elevado a los curanderos indígenas “a la dignidad de médicos y organizados en un orden que ha sido puesto en condiciones de igualdad con el de los practicantes de la medicina llamada occidental”^73^.

Más aún, María José Araya, al describir y analizar la situación de las parteras empíricas, a través de la trayectoria del Consejo de Organizaciones de Médicos y Parteras Indígenas Tradicionales de Chiapas (COMPITCH), considera que la visión de la falta de agencia en las parteras es producto de una visión externa a ellas ya que, desde el punto de vista de estas curadoras, ellas no están subordinadas, evidencian capacidad de resistencia, y siguen manteniendo sus lógicas culturales. Si bien reconoce que inicialmente el COMPITCH y otras organizaciones fueron financiadas y orientadas por el Instituto Nacional Indigenista, son cada vez más autónomas a partir del período 1998-2000^58^. 

También se observa que, en varios grupos étnicos, las parteras se niegan a atender partos en los servicios de salud^74^; mientras que una parte de los curadores tradicionales organizados proponen una participación autónoma respecto de la biomedicina^54^, lo cual es correcto en lo referente a que una parte de las parteras se nieguen a trabajar con el sector salud, pero no lo es en términos de la existencia de organizaciones y movimientos que enfrenten al sector salud. En México, no observamos luchas culturales ni políticas, y menos aún organizaciones para enfrentar la expansión biomédica en los pueblos originarios, pero tampoco para defender a la medicina tradicional.

Pero, además, salvo en el caso de la brujería, los estudios de medicina tradicional no han descripto ni analizado los procesos y funciones de control sociocultural que cumple medicina tradicional, aunque frecuentemente hacen referencia a las funciones de control social desarrolladas por la biomedicina. Y lo que necesitamos asumir, es que en todas las sociedades de las que tenemos información, los procesos de salud-enfermedad-atención-prevención constituyen uno de los principales campos de control sociocultural y, especialmente, en las sociedades nativas en las que gran parte de las enfermedades son generadas por la transgresión a las normas sociales y culturales, que necesitan ser “curadas” por un terapeuta tradicional. Pero la reducción constante de curadores tradicionales, sobre todo de los “brujos” y chamanes, así como su medicalización y subordinación biomédica conduce a la reducción o eliminación de controles socioculturales, y a que por lo menos una parte sea desarrollada por la biomedicina. Esto, entre otras cuestiones, nos lleva a preguntarnos sobre ¿qué tipo de cosmovisiones dominan actualmente en los diferentes pueblos originarios?

En términos más o menos históricos, las relaciones entre biomedicina y medicina tradicional fueron pensadas inicialmente como aculturación/transculturación. Harman sostiene que “en las prácticas médicas entre los mayas del altiplano, la fusión y no la sustitución es el más importante proceso de cambio”^13^, concluyendo que la cambiante cultura indígena se caracteriza por “la aceptación de formas occidentales reinterpretadas de acuerdo a los principios existentes basados en causas mágico/religiosas de las enfermedades”^13^. Cuarenta años después, Meneses^29^ sostiene que, en dicho altiplano se da un sincretismo y complementariedad entre la medicina tradicional y la biomedicina.

En general, los analistas tratan de demostrar que los sujetos y grupos étnicos no son marionetas supeditadas a los proyectos de los actores y fuerzas sociales dominantes, lo que considero correcto, siempre que asumamos que dichos grupos subalternos pueden aculturarse y asimilarse como necesidad pragmática de supervivencia. En las transacciones entre medicina tradicional y biomedicina, los grupos subalternos se apropian y utilizan lo que necesitan de la biomedicina para solucionar o reducir sus problemas de salud, y lo pueden hacer dándoles significados propios o usando los generados por la propia biomedicina. Y ello más allá del peso de sus cosmovisiones^75^.

Si bien el saber hegemónico se ha apropiado de un discurso que reconoce y valora las diferencias para desarrollar su hegemonía, esto se hace a partir de las condiciones de vida, de los pueblos originarios, por lo que no considero que haya que lamentar y conjurar las pérdidas y/o resignificaciones culturales, sino tratar de ver qué hacen con ellas no solo los que hegemonizan, sino los subalternizados. Y el pragmatismo de los sectores subalternos los lleva a usar y apropiarse de la biomedicina no solo por aculturación, sino como mecanismo de supervivencia. Es en gran medida por esto, que analistas como Pedersen dudan de que se pueda producir una integración en America Latina entre medicina tradicional y biomedicina, dado que “en la mayoría de los países, las propuestas de integración provienen de los sectores dominantes de la medicina oficial, sin tener en cuenta las opiniones o actividades de los propios practicantes de la medicina tradicional”^67^. Lo que también cuestiona Freyermuth Enciso al sostener que “no es aceptable que las políticas encaminadas a la interrelación pretendan incluir a los terapeutas indígenas en programas que les son ajenos, como igualmente difícil sería pretender que los médicos aprendieran y utilizaran como elementos curativos la herbolaria, los ritos y los rezos”^53^. Pero, pese a estos cuestionamientos, el sector salud ha “integrado” a los curadores tradicionales, convirtiéndolos en varias experiencias en auxiliares de salud, o medicalizando y finalmente excluyendo el trabajo de las parteras. 

Mientras algunos analistas recuperan el uso de recursos tradicionales o por lo menos de auxiliares de salud, remitiendo a la experiencia china de los médicos descalzos, otros analistas, como Juan César García^76^ consideran que esta es una estrategia colonialista, e incluso sostiene que muchas de las ideas de los sanitaristas que impulsan este recurso humano han sido inspiradas por las acciones coloniales, lo que no ocurriría si los procesos de integración estuvieran manejados con autonomía por los sectores subalternos^33^.

Ahora bien, en términos teóricos e ideológicos, así como en términos explícitos e implícitos, los estudios sobre las sociedades indígenas, incluida la medicina tradicional, han focalizado e impulsado casi exclusivamente los procesos culturales, incluso planteando la necesidad de impulsar el control cultural, lo cual fue desarrollado en forma teórica -aunque no etnográfica- por Bonfil Batalla^77^^,^^78^. Es necesario señalar que esta orientación culturalista contrasta con lo que considero el principal estudio etnográfico realizado por Bonfil Batalla, y me refiero al *Diagnóstico sobre el hambre en Sudzal, Yucatán*^79^, en el que sin dejar de tomar en cuenta los aspectos culturales, las condiciones dominantes refieren a procesos sociales y económico políticos^80^.

Este control cultural refiere a sociedades que están en vías de cambio, a sociedades nativas en las que las y los jóvenes ya no tienen que ver con lo que piensan sus padres y abuelos. Las y los jóvenes ya no piensan en términos de sus cosmovisiones, lo que es analizado por Page respecto de los tzotziles de Chiapas^81^^,^^82^. Y si bien Bonfil Batalla reconoce el cambio y propone un control cultural basado en los saberes de los sectores subalternos y en su autonomía, al reducir todo a lo cultural propicia que en México, en los estudios sobre medicina tradicional, domine un culturalismo que reduce el poder y lo económico a lo cultural, lo que ha sido cuestionado especialmente por Susana Ramírez Hita, quien sostiene que:

La salud intercultural termina por ser una estrategia política y económicamente barata para hacer visibles cambios y mantener a los sectores subalternos apaciguados, proponiéndoles una revalorización de su cultura a través del respeto a sus medicinas tradicionales.^64^

Esto puede darse o no, pero al ser manejado por el Estado y no por los sectores subalternos, tiende a imponer las políticas “culturales” de los sectores dominantes.

Walsh^33^ sostiene argumentos similares, pero subraya que la interculturalidad tiene por objetivo construir un Estado plurinacional, una nueva democracia anticolonialista, anticapitalista y antiimperialista, y aclara que entiende la interculturalidad no como un concepto que refiere al contacto y conflicto entre occidente y otras civilizaciones, sino que supone partir del pensamiento y experiencia india, que implica conocer el pensamiento occidental que posibilita construir un pensamiento “otro”^33^. Esta perspectiva no implica reducir todo a lo cultural, sino proponer pensar y actuar a traves de lo cultural y de lo económico-político, lo que en cierta medida se desarrolló en México con el Ejército Zapatista de Liberación Nacional (EZLN), así como en Ecuador, Bolivia y últimamente en Chile. En este país las organizaciones indígenas buscan el reconocimiento político, ya sea para su inclusión en la democracia o para permitir su desarrollo autonómico en esferas como el territorio, la cultura, la economía^83^. Esto ha sido fundamentado a nivel de las organizaciones indígenas mapuches y, especialmente, respecto de los procesos de salud-enfermedad-atención-prevención por Andrés Cuyul Soto, quien parte de la autonomía social, cultural, política y territorial de los mapuches, pero sin rechazar la biomedicina, a la que no ve como una penetración occidental, sino que la analiza como parte de la vida indígena, propiciando el desarrollo de la medicina tradicional nativa^22^^,^^23^.

De lo analizado surgen toda una serie de procesos que contribuyen a entender no solo la expansión biomédica, sino su demanda, uso y apropiación por parte de los pueblos originarios. La eficacia biomédica -evidenciada por lo menos parcialmente en varios aspectos nucleares como la reducción de las tasas de mortalidad, la ampliación de la esperanza de vida, la reducción de las muertes “evitables”- es reconocida por los grupos nativos, al igual que el incremento de los servicios biomédicos en zonas indígenas, más allá de sus déficits, y el uso simultáneo de medicina tradicional y biomedicina sin problemas de incompatibilidad. Nuestra experiencia de investigación en varias zonas de México coincide con lo señalado por Kelly y Carrera para los yanomami de Venezuela:

En la gran mayoría de las intervenciones biomédicas de campo, las divergencias entre la concepción yanomami y biomédica de la enfermedad son poco relevantes. Los pacientes acuden al ambulatorio, se nebulizan, etc. Es solo cuando los pacientes evolucionan a estados de gravedad y la sospecha de muerte… que el paciente suele abandonar el ambulatoirio y busca tratamiento shamánico.^84^

Y esto, en el caso de México, ocurre cuando las enfermedades graves refieren a problemas de salud mental dado que, en el caso de las enfermedades graves físicas, se demanda cada vez más la atención biomédica. Pero, además, frente a los problemas como el desarrollo de pandemias, es la biomedicina y no la medicina tradicional la que propone acciones y productos que pueden enfrentarlos, y que son reconocidos a nivel internacional. Frente al VIH-sida, el virus del Ébola o la pandemia de covid-19 no fue la medicina tradicional la que se potenció y actuó a nivel nacional e internacional, sino la biomedicina. Más aún, cada vez más se espera que la solución o reducción de los problemas de salud sea generada por la biomedicina, lo que es avalado reiteradamente por la OMS al informar, por ejemplo, en 2017, que los avances sin precedentes en la lucha contra las enfermedades tropicales desatendidas^85^. Correlativamente, como ya hemos señalado, no contamos con estudios que evidencien la eficacia de la medicina tradicional, pues no suelen presentar datos específicos.

La autoatención constituye el proceso que en toda sociedad integra las diferentes formas de atención vigentes a nivel local^86^ y pone en evidencia que, en los grupos originarios, las acciones, concepciones y productos biomédicos forman parte cada vez más importante de “su” forma de autoatención. La población necesita apropiarse de un saber médico que evidencia mayor eficacia, y lo hace en gran medida a través de la autoatención. Y es en parte por esto que la biomedicina tiende a colocar la responsabilidad/culpabilidad respecto de los tratamientos clínicos en el sujeto y su familia. Y lo hace a través de reglas técnicas que tratan de imponer profesionalmente las instituciones biomédicas. Pero estas reglas operan dentro de relaciones en las que los dos actores sociales construyen sistemas de expectativas similares y diferenciales respecto del “otro”. 

La biomedicina, como ya lo describimos, se expande en forma directa e indirecta a través de actividades e instituciones, no se expande en forma aislada, sino en forma complementaria con la expansión de procesos económicos, educativos, religiosos que se refuerzan mutuamente. Pero además, la biomedicina se expande también a través de la medicina tradicional y de algunas medicinas alternativas y complementarias dado que, por un lado, van formando parte de las formas de atención de los pueblos originarios y, por otro, los sectores sociales medios altos y altos se apropian de formas de atención tradicionales y alternativas dentro de espacios escenográficos como el del llamado parto humanizado^49^, o de otros espacios que forman parte de un turismo sanador y especialmente chamánico^87^^,^^88^.

Lo que necesitamos reconocer es que la biomedicina se expande para imponer sus saberes, pero que el núcleo de la expansión no consiste en eliminar a la medicina tradicional, ni en controlar a los sujetos y las sociedades, ya que estas surgen funcionalmente durante el proceso de expansión. La biomedicina se expande a través de actividades clínicas y preventivas, que pueden ser impuestas con violencia profesional, mientras que otras actividades y productos biomédicos serán utilizados por los sectores subalternos por su eficacia, subrayando que el conjunto de los procesos de salud-enfermedad-atención-prevención opera dentro de relaciones de hegemonía/subalternidad, pero con escaso desarrollo de contrahegemonía por parte de los sectores subalternos^3^^,^^4^^,^^89^.

Esto no niega que a través de la medicina tradicional puedan generarse fuertes procesos contrahegemónicos, como lo desarrolló Fanon^90^^,^^91^ para los sectores sociales subalternos argelinos, pero que no observamos en el caso de la medicina tradicional mexicana, por lo menos a nivel de los pueblos originarios, aunque sí podemos encontrar propuestas de antropólogos y de otros profesionales e intelectuales que formulan consignas contrahegemónicas, pero que no operan en la realidad. Más aún, no solo no hemos observado movimientos o acciones contrahegemónicas en los curadores tradicionales, sino que tampoco hemos detectado que la medicina tradicional sea un reclamo básico de los movimientos étnicos; lo cual, obviamente, no ignora la existencia intermitente de movilizaciones, enfrentamientos y transacciones de los pueblos indios sobre todo en Bolivia, Ecuador y Chile, pero sin que la defensa y/o proyección de la medicina tradicional constituya un aspecto clave de dichas movilizaciones y transacciones.
